# Apatinib suppresses lung cancer stem-like cells by complex interplay between β-catenin signaling and mitochondrial ROS accumulation

**DOI:** 10.1038/s41420-021-00480-6

**Published:** 2021-05-12

**Authors:** Jianyun Zhu, Xiaoting Li, Chunhua Liang, Xu Zhou, Miaomiao Ge, Yue Chen, Jianliang Jin, Juan Yin, Haie Xu, Chunfeng Xie, Caiyun Zhong

**Affiliations:** 1 Department of Laboratory, The Affiliated Suzhou Hospital of Nanjing Medical University, Suzhou Municipal Hospital, Gusu School, Nanjing Medical University, Suzhou, Jiangsu 215008 China; 2 Department of Nutrition and Food Safety, School of Public Health, Nanjing Medical University, Nanjing, Jiangsu 211166 China; 3 Research Centre for Bone and Stem Cells, Department of Human Anatomy, Key Laboratory for Aging & Disease, Nanjing Medical University, Nanjing, Jiangsu 211166 China; 4 Cancer Research Division, Center for Global Health, School of Public Health, Nanjing Medical University, Nanjing, Jiangsu 211166 China

**Keywords:** Non-small-cell lung cancer, Cancer stem cells

## Abstract

The abnormal activation of Wnt/β-catenin signaling plays a critical role in the development of lung cancer, which is also important in the generation and maintenance of lung cancer stem cell (CSC). CSCs have unique capabilities to resist anticancer therapy, seed recurrent tumors, and disseminate to and colonize distant tissues. Apatinib, a small-molecule VEGFR2-tyrosine kinase inhibitor, shows highly efficient antitumor activity in heavily treated, chemoresistant, and metastatic lung cancer. We speculated that inhibition of Wnt/β-catenin signaling and targeting lung CSCs could be one of the anti-tumor mechanisms of apatinib. In the present study we demonstrated that apatinib repressed lung CSC-like traits by hindering sphere formation ability, lung CSC-related marker expression and decreasing chemoresistance derived stemness. Mechanistically, apatinib exerted its anti-CSC effects by inhibiting β-catenin and its downstream targets. Moreover, apatinib induced the production of reactive oxyen species (ROS), which participated in the inhibitory effects of apatinib on lung CSCs. It was found that β-catenin regulated apatinib-induced production of ROS. Inhibition or promotion of ROS production with N-acetyl-L-cysteine or H_2_O_2_ not only upregulated or downregulated β-catenin expression, but also prevented or promoted DNA damage, rescued or impeded sphere formation, respectively. Collectively, our findings reveal that apatinib directly inhibits β-catenin signaling and promotes ROS generation to suppress lung CSC-like characteristics. A clearer understanding of the anti-cancer mechanisms of apatinib is required for its better application in combating advanced and refractory/recurrent lung cancer when combined with conventional chemotherapy.

## Introduction

Lung cancer is the most common cancer in China worldwide, resulting in ≈1.8 million deaths globally in 2018^1^. Non-small cell lung cancer (NSCLC) is the main histological type, representing ≈85% of all lung cancer cases^2^. Most NSCLC patients are diagnosed at advanced stage and have a poor prognosis, with few long-term survivors. Despite the continued development of newer therapies with novel mechanisms of action, reshaping the treatment paradigm of NSCLC over the last two decades, the overall survival rates of NSCLC remain low. Therefore, a search of new drugs and combination therapies is needed to expand clinical benefits, improve the current therapy and reduce the mortality in NSCLC.

Numerous studies have demonstrated that lung cancer is a highly heterogeneous disease and contains cancer stem cells (CSCs) that possess the ability to self-renew and generate heterogeneous lineages of other cell types. CSCs characteristics, including drug resistance and enhanced migration, have become targets for cancer therapy. Lung CSCs express specific surface markers, including Nanog, Oct4, Sox2, CD44, and CD133^3^, and have been isolated from NSCLC cell lines^4^ and patients. Lung CSCs also display high aldehyde dehydrogenase (ALDH1) activity, which is positively associated with aggressive biological behavior and a poor prognosis of NSCLC^5^. Another hallmark of CSCs that distinguishes them from most other cancer cells is that they exhibit a multi-drug resistance phenotype by overexpression of drug efflux transporters on the cell surface, including ABCB1/P-gp, ABCC1/MRP1, and ABCG2/BCRP^6^. The increased expression of drug efflux transporters closely correlates with the CSC-like phenotype and chemoresistance of lung carcinomas^7^. Thus, new drugs targeting these specific lung cancer markers may effectively suppress lung CSCs and overcome drug resistance to achieve better therapeutic efficacy.

The canonical Wnt signaling pathway has always been a prevalent theme in cancer biology and is responsible for the progression in several types of human malignancies^8^. Notably, the hyperactivation of Wnt-mediated signaling is closely associated with lung cancer “stemness” and chemoresistance^9–11^. Upon Wnt activation, the accumulated β-catenin in the cytoplasm translocates to the nucleus, and subsequently activates the transcription of β-catenin-responsive genes, such as *MYC*^12^ and *CCND1*^13^. Many studies have emphasized the role of over-activated β-catenin in the regulation of lung CSCs. Given that mutations in β-catenin are uncommon in NSCLC^14^, new drugs that target Wnt/β-catenin signaling pathway and inhibit lung CSCs may be of particular interest.

Interestingly, ROS can induce resistance to therapy at both high and low concentrations. Many studies have found that CSCs possess a highly compatible ROS scavenging system, which maintains them at lower levels of ROS than more mature cancer cells and resists the oxidative stress induced by radio- and chemotherapy. Therefore, the disruption of this system may be an effective strategy to decrease or eliminate CSCs.

Apatinib, a potent vascular endothelial growth factor receptor-2 (VEGFR-2)- tyrosine kinase inhibitor (TKI), exerts antiangiogenic and antineoplastic functions, and has promising efficacy and acceptable toxicity profile for the treatment of solid tumors^15–17^. Notably, an increasing number of studies have found that apatinib monotherapy or combined chemotherapy has been shown as an effective treatment for a variety of cancers, particularly for advanced and refractory NSCLCs^18–20^. For example, in epidermal growth factor receptor (EGFR)-mutant NSCLC, combination of apatinib and gefitinib enhanced the antitumor efficacy in comparison to EGFR-TKIs alone, and delayed the onset of treatment resistance^21,22^. However, the underlying precise mechanisms are not fully understood. In this study, we used lung cancer spheroids as in vitro model to investigate the potential impact of apatinib on lung cancer stem-like cells. We found that apatinib treatment significantly reduced the ability of sphere formation and the expression of lung CSC-specific markers. Moreover, apatinib treatment directly inhibited β-catenin signaling, and promoted ROS generation by disturbing redox balance and mitochondrial membrane potential, resulting in the suppression of lung CSC-like traits. Thus, an in-depth understanding of the anti-cancer mechanism of apatinib in the treatment of lung cancer is important for optimizing treatment strategies.

## Results

### Apatinib inhibited lung CSC-like properties

**Fig. 1:**
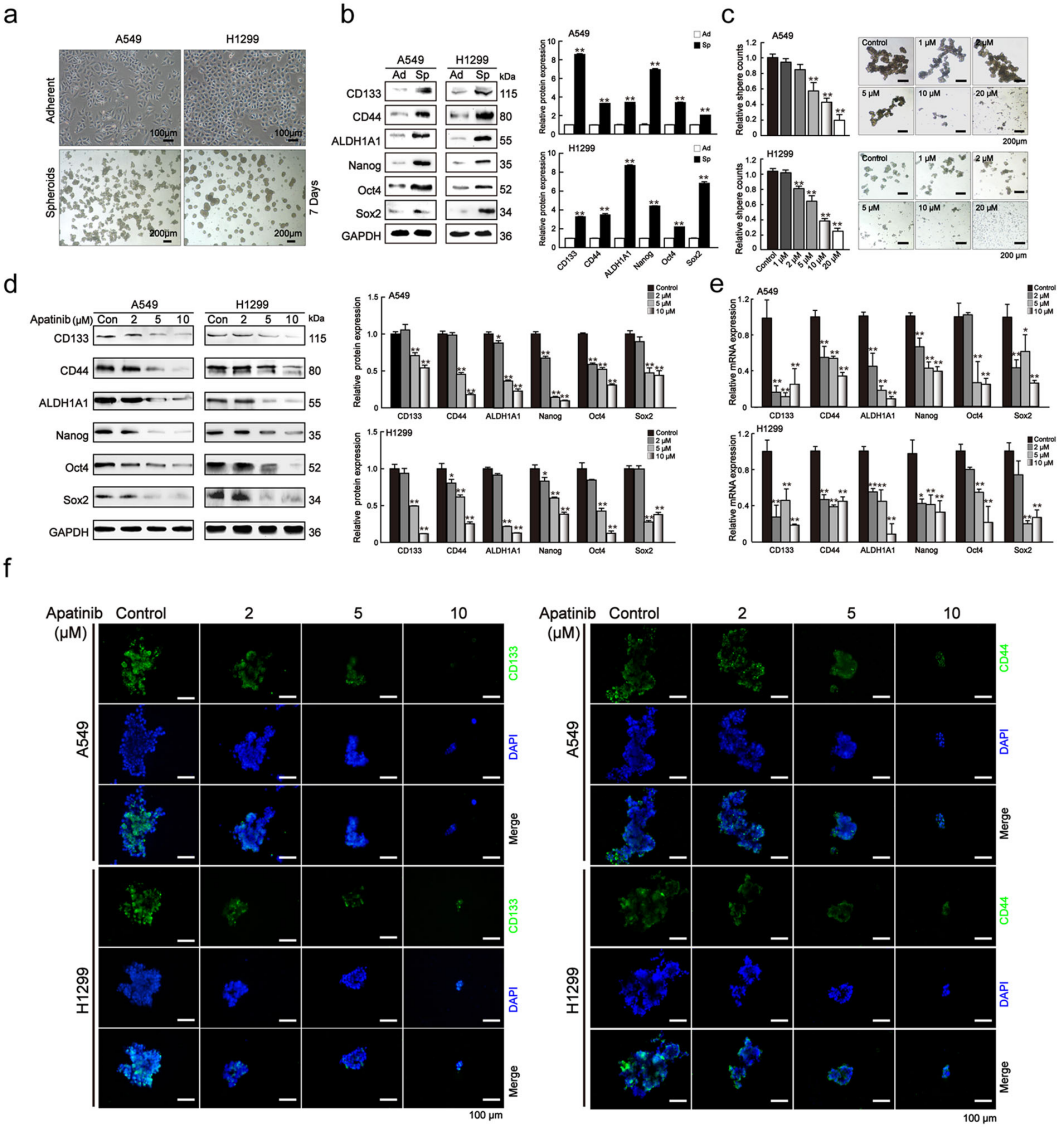
Apatinib inhibited lung CSC-like properties. **a** Sphere-forming capacities of A549 and H1299 cells. Scale bar = 100 μm in adherent group, scale bar = 200 μm in spheroids group. **b** Protein levels of lung CSC-related markers (CD133, CD44, and ALDH1A1) and stemness-related genes (Nanog, Oct4, and Sox2) in adherent cells and spheroids on day 7. Ad adherent cells, Sp spheroids. Unpaired *t*-test was used. Data are presented as mean ± SD (*n* = 3). **p < 0.01 compared to the control group. **c** Sphere-forming capacities of A549 and H1299 cells treated by apatinib at various concentrations (0, 1, 2, 5, 10, and 20 μM) on day 7. Scale bar = 200 μm. One-way ANOVA (Bonferroni’s multiple-comparison test) was used. Data are presented as mean ± SD (*n* = 3). **p < 0.01 compared to the control group. **d** Protein levels of lung CSC markers in spheroids on day 7 after apatinib treatment. One-way ANOVA (Bonferroni’s multiple-comparison test) was used. Data are presented as mean ± SD (*n* = 3). *p < 0.05, **p < 0.01 compared to the control group. **e** mRNA expression of lung CSC markers in spheroids on day 7 after apatinib treatment. One-way ANOVA (Bonferroni’s multiple-comparison test) was used. Data are presented as mean ± SD (*n* = 3). *p < 0.05, **p < 0.01 compared to the control group. **f** Representative immunofluorescence images of CD133 (green) and CD44 (green) in apatinib-treated spheroids. DAPI: blue. Scale bar = 100 μm.

A large amount of clinical data indicates that apatinib monotherapy or combined chemotherapy has promising efficacy for the treatment of advanced NSCLC after the failure of chemotherapy or other targeted therapies^22,23^, and even preferentially acts on drug-resistant lung cancer cells^24^, suggesting that apatinib may significantly affect the stemness of lung CSCs. Therefore, we examined the effect of apatinib on lung CSCs stemness using a sphere-forming assay. Our previous studies have shown that lung cancer cells could form spheroids in a specific stem cell culture system, which exhibited lung CSC-like properties^25^. Figures 1a and b showed that A549 and H1299 cells formed stable spheroids in serum-free medium (SFM) and expressed high levels of lung CSC-specific markers, including lung CSC-related markers (CD133, CD44, ALDH1A1) and stemness-related genes (Nanog, Oct4 and Sox2). Spheroids were cultured with or without various concentrations of apatinib. Figure 1c, showed apatinib significantly reduced the number and size of tumorspheres. Moreover, 20 μM apatinib markedly inhibited the formation of spheres, i.e., less than 30% of that formed in the control cells (Fig. 1c).

Based on the above observation that apatinib inhibited sphere formation, we reasoned that apatinib might inhibit the expression of lung CSC-specific markers, which promote the stemness of CSCs^26^. Our data showed that apatinib dramatically suppressed the protein expression of CD133, CD44, ALDH1A1, Nanog, Oct4, and Sox2 (Fig. 1d). Simultaneously, consistent changes were observed in the mRNA expression of these markers in both spheroids (Fig. 1e). A similar decrease in CD133 and CD44 expression with apatinib treatment was also revealed by immunofluorescence staining (Fig. 1f). Taken together, these results demonstrated that apatinib efficiently inhibited lung CSC-like properties by downregulating sphere formation ability and lung CSC-specific marker expression.

### Apatinib decreased drug resistance-related genes

**Fig. 2:**
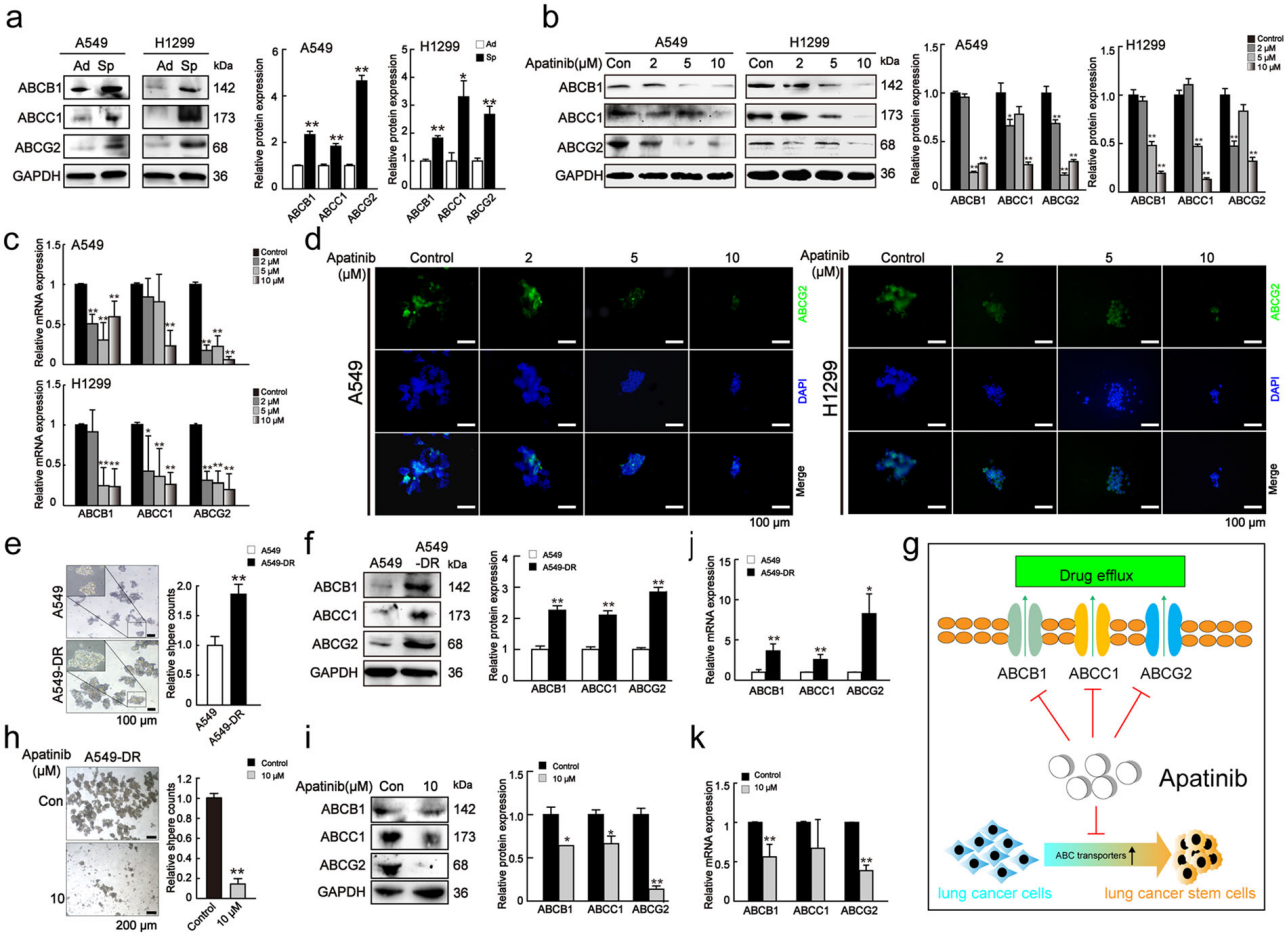
Apatinib decreased drug resistance-related genes. **a** Protein levels of drug resistance-related genes (ABCB1, ABCC1, and ABCG2) in adherent cells and spheroids. Ad adherent cells, Sp spheroids. Data are presented as mean ± SD (*n* = 3). *p < 0.05, **p < 0.01 compared to the control group. **b** Protein levels of drug resistant-related genes in spheroids on day 7 after apatinib treatment. One-way ANOVA (Bonferroni’s multiple-comparison test) was used. Data are presented as mean ± SD (*n* = 3). *p < 0.05, **p < 0.01 compared to the control group. **c** mRNA expression of drug resistant-related genes in spheroids on day 7 after apatinib treatment. One-way ANOVA (Bonferroni’s multiple-comparison test) was used. Data are presented as mean ± SD (*n* = 3). *p < 0.05, **p < 0.01 compared to the control group. **d** Representative immunofluorescence images of ABCG2 (green) in apatinib-treated spheroids. DAPI: blue. Scale bar = 100 μm. **e**. Sphere-forming capacities of A549 and CDDP-resistant A549 cells (A549-DR) on day 7. Scale bar = 100 μm. Data are presented as mean ± SD (*n* = 3). Unpaired *t*-test comparisons were used. **p < 0.01 compared to the A549 group. **f**, **j** Protein and mRNA levels of drug resistance-related genes in A549 and A549-DR cells on day 7. One-way ANOVA (Bonferroni’s multiple-comparison test) was used. Data are presented as mean ± SD (*n* = 3). *p < 0.05, **p < 0.01 compared to the A549 group. **h** Sphere-forming capacities of A549-DR cells after 10 μM apatinib treatment on day 7. Scale bar = 200 μm. Unpaired *t*-test was used (*n* = 3). Data are presented as mean ± SD. **p < 0.01 compared to the control group. **i**, **k** Protein and mRNA levels of drug resistance-related genes in A549-DR cells with or without 10 μM apatinib treatment on day 7. Unpaired *t*-test was used. Data are presented as mean ± SD (*n* = 3). *p < 0.05, **p < 0.01 compared to the control group. **g** Schematic model showing the inhibition of apatinib on drug resistance genes.

Because the development of chemotherapy resistance leads to the failure of cancer treatment, which is also related to the CSCs property, we next examined whether apatinib inhibited the expression of resistance-related genes that are highly expressed in CSCs, especially ABC transporters^27^. We showed that both A549 and H1299 spheroids expressed higher protein levels of ABC transporters (ABCB1, ABCC1, and ABCG2) when compared to the corresponding adherent cells (Fig. 2a). Next, we showed that apatinib reduced the protein and mRNA expression of these ABC transporters in both spheroids (Figs. 2b and c), which was consistent with the previous studies^28^. Immunofluorescence staining also demonstrated that apatinib downregulated ABCG2 level (Fig. 2d).

To further confirm the above findings, we also examined whether apatinib had a similar effect on cisplatin (CDDP)-resistant A549 cells which also display a stem-like signature^29^. Our results showed a significant increase in sphere formation and the expression of drug resistance-related genes (ABCB1, ABCC1, and ABCG2) in the cisplatin-resistant cells (A549-DR) relative to A549 cells (Figs. 2e, f, and j). 10 μM apatinib dramatically reduced sphere formation and the protein and mRNA expressions of ABCB1 and ABCG2 in A549-DR cells (Figs. 2h, i, and k). Taken together, these data showed that apatinib disturbed therapy resistance to suppress the stemness of lung CSCs (Fig. 2g).

### Apatinib reduced β-catenin and β-catenin-responsive genes

Among the pathways involved in the development and maintenance of CSCs, overactivation of the Wnt/β-catenin pathway is widely considered to be one of the most frequent events in a variety of tumor types^30^. There is solid evidence that lung CSC characteristics are maintained by the activation of β-catenin and upregulation of its responsive gene^11^. Our previous studies also demonstrated that targeting Wnt/β-catenin pathway in lung CSCs could be a promising strategy for cancer therapy^25^.

**Fig. 3:**
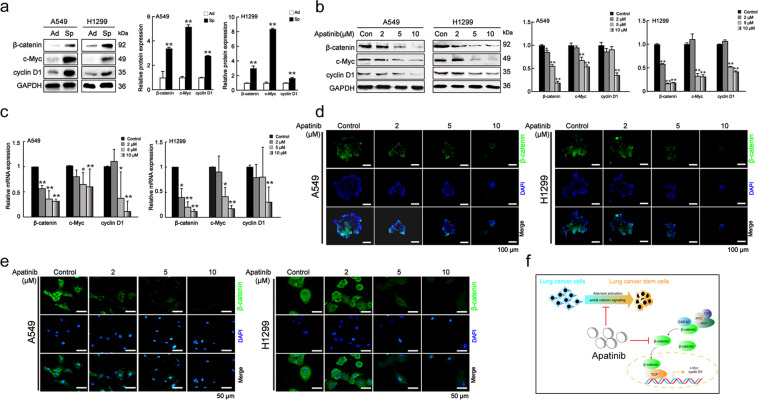
Apatinib reduced β-catenin expression and downregulated its responsive genes. **a** Protein levels of β-catenin and downstream genes (c-Myc and cyclin D1) in adherent cells and spheroids. Ad adherent cells, Sp spheroids. Unpaired *t*-test was used. Data are presented as mean ± SD (*n* = 3). **p < 0.01 compared to the control group. **b** Protein and mRNA levels of β-catenin and downstream genes in A549 and H1299 spheroids on day 7 after apatinib treatment. One-way ANOVA (Bonferroni’s multiple-comparison test) was used. Data are presented as mean ± SD (*n* = 3). *p < 0.05, **p < 0.01 compared to the control group. **c** mRNA levels of β-catenin and downstream genes in spheroids on day 7 after apatinib treatment. One-way ANOVA (Bonferroni’s multiple-comparison test) was used. Data are presented as mean ± SD (*n* = 3). *p < 0.05, **p < 0.01 compared to the control group. **d** Representative immunofluorescence of β-catenin (green) in apatinib-treated spheroids. DAPI: blue. Scale bar = 100 μm. **e** Representative immunofluorescence images of β-catenin (green) in adherent cells on day 2 after apatinib treatment. DAPI: blue. Scale bar = 50 μm. **f** Schematic representation of the effect of apatinib on β-catenin signaling in lung cancer.

Based on these observations, we investigated whether apatinib could exert its effect on lung CSCs by inhibiting Wnt/β-catenin pathway. The protein levels of β-catenin and its responsive genes (c-Myc and cyclin D1) were highly elevated in A549 and H1299 sphere-forming cells in comparison with the adherent cells (Fig. 3a). After apatinib treatment, there was a significant decrease in the protein and mRNA expressions of β-catenin, c-Myc and cyclin D1 (Fig. 3b–d). Immunofluorescence staining further demonstrated that apatinib also reduced β-catenin expression in adherent cells (Fig. 3e). These data suggested that apatinib suppressed Wnt/β-catenin pathway (Fig. 3f).

### β-catenin regulated CSC-like characteristics and drug resistance in lung cancer cells

**Fig. 4:**
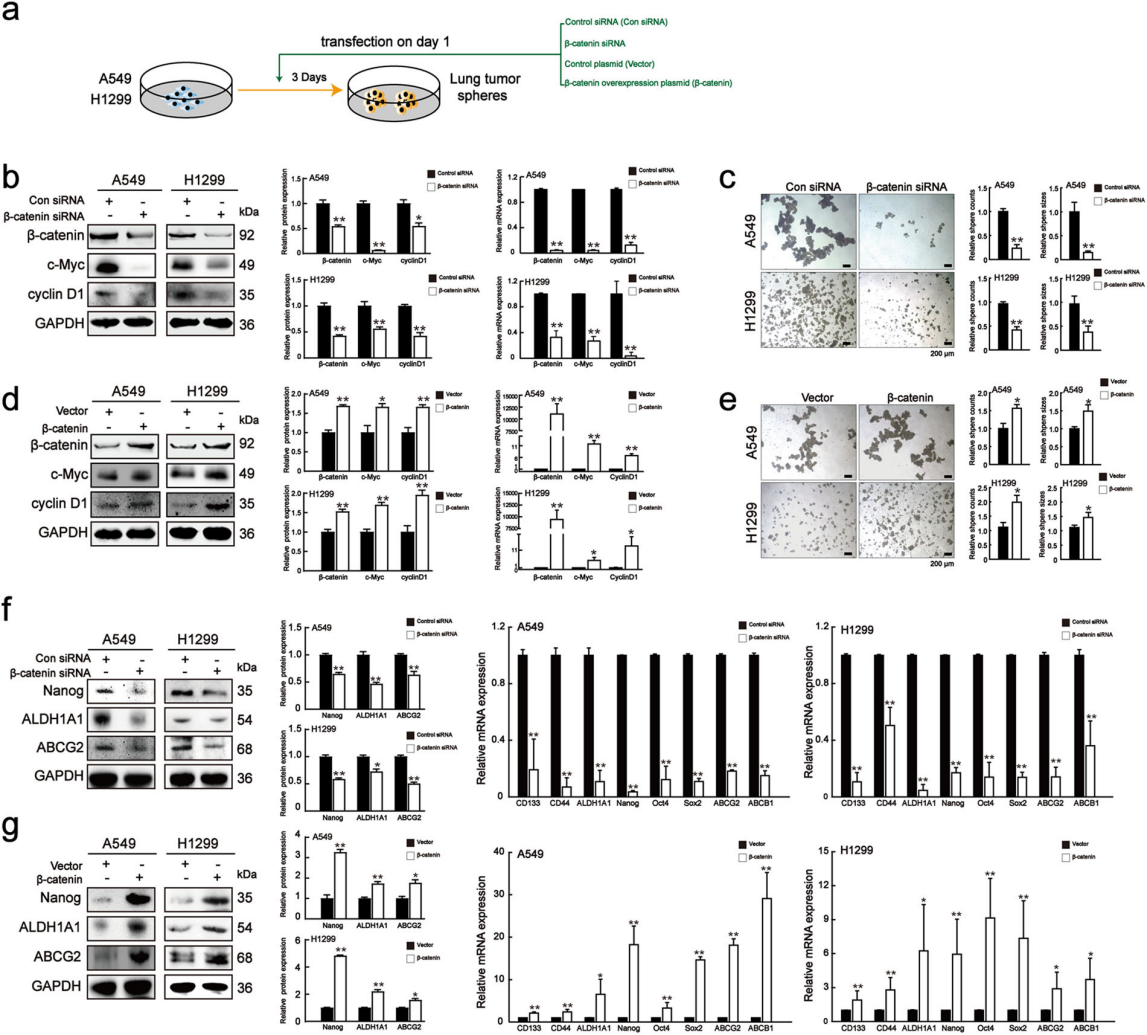
β-catenin regulated lung CSC-like characteristics and drug resistance. **a** Schematic diagram of lung tumor spheres culture after transfection. A549 and H1299 cells were transfected with con siRNA, β-catenin siRNA, control vector, and pcDNA-β-catenin. **b** Protein and mRNA levels of β-catenin, c-Myc, and cyclin D1 in spheroids after being transfected with control siRNA and β-catenin siRNA. Unpaired *t*-test was used. Data are presented as mean ± SD (*n* = 3). *p < 0.05, **p < 0.01 compared to the con siRNA group. **c** Sphere-forming capacities of cells transfected with control siRNA and β-catenin siRNA. Scale bar = 100 μm. Unpaired *t*-test was used. Data are presented as mean ± SD (*n* = 3). **p < 0.01 compared to the con siRNA group. **d** Protein and mRNA levels of β-catenin, c-Myc and cyclin D1 in spheroids after transfected with control vector and pcDNA-β-catenin. Unpaired t-test was used. Data are presented as mean ± SD (*n* = 3). *p < 0.05, **p < 0.01 compared to the vector group. **e** Sphere-forming capacities of cells transfected with control vector and pcDNA-β-catenin. Scale bar = 100 μm. Unpaired *t*-test was used. Data are presented as mean ± SD (*n* = 3). *p < 0.05 compared to the vector group. **f** Protein and mRNA levels of lung CSC markers and drug resistance genes in spheroids after being transfected with control siRNA and β-catenin siRNA. Unpaired *t*-test was used. Data are presented as mean ± SD (*n* = 3). *p < 0.05, **p < 0.01 compared to the con siRNA group. **g** Protein and mRNA levels of lung CSC markers and drug resistance genes in spheroids after being transfected with control vector and pcDNA-β-catenin. Unpaired *t*-test was used. Data are presented as mean ± SD (*n* = 3). *p < 0.05, **p < 0.01 compared to the vector group.

To further characterize the role of β-catenin in regulating lung CSCs, we used β-catenin siRNA or pcDNA-β-catenin to downregulate or upregulate β-catenin expression in A549 and H1299 cells, and evaluated its impact on stemness (Fig. 4a). The transfection efficiency of β-catenin was confirmed by western blot and qRT-PCR analyses, respectively, through comparison with that of a negative control (Figs.4b and d). As shown in Fig. 4b, β-catenin knockdown effectively suppressed the expressions of β-catenin, c-Myc, and cyclin D1. In contrast, β-catenin overexpression had the opposite effect (Fig. 4d). The sphere-forming assay showed that β-catenin knockdown resulted in smaller and less tumor spheres, while β-catenin overexpression formed more and larger spheres (Figs. 4c and e). Furthermore, β-catenin knockdown downregulated lung CSC-specific markers and drug resistance-related genes, while β-catenin overexpression had the opposite effects (Figs. 4f and g). Taken together, these results indicated that β-catenin promoted lung CSC-like properties and drug resistance.

### The inhibitory effects of apatinib on lung CSC-like properties were mediated by β-catenin

**Fig. 5:**
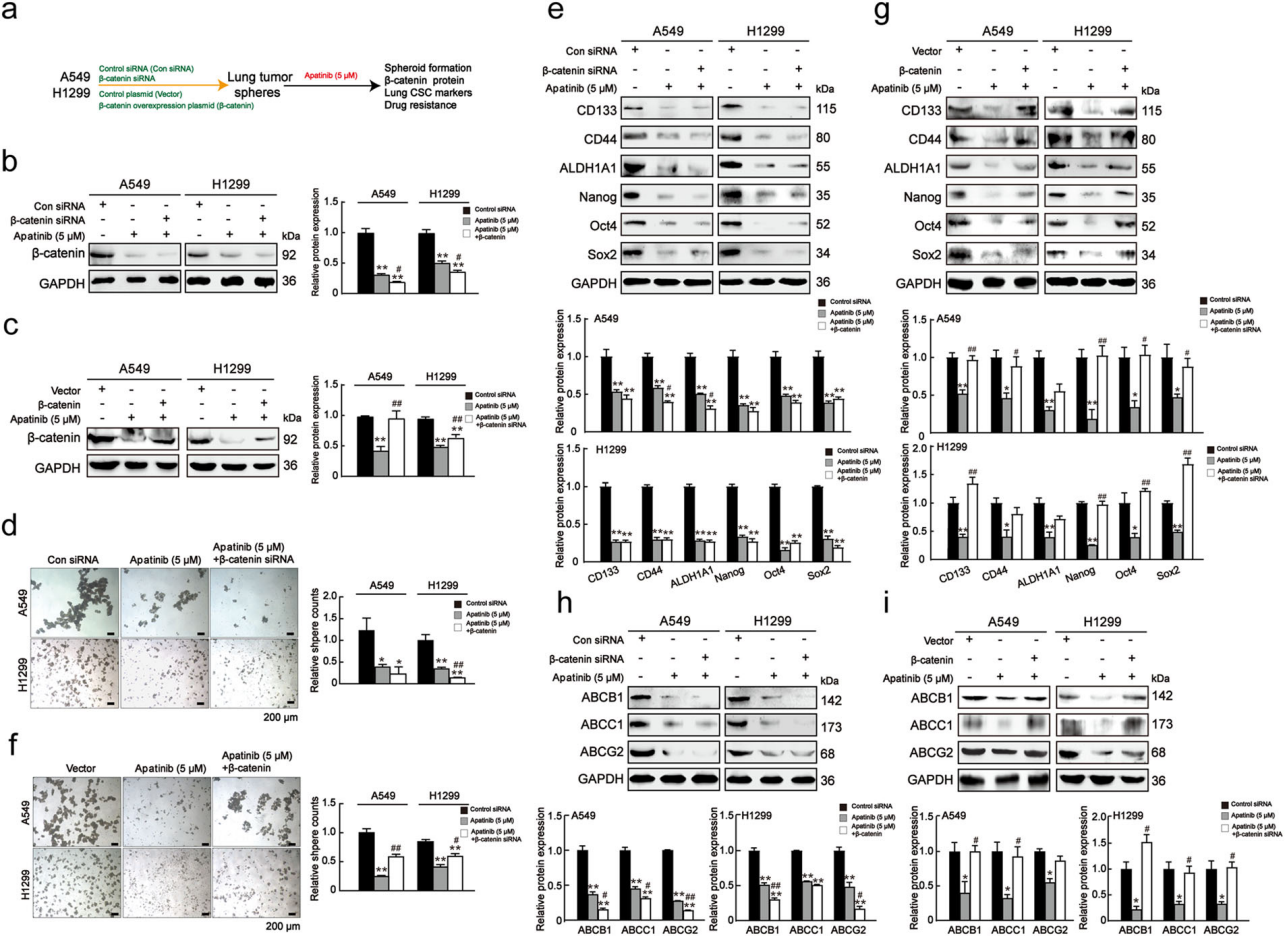
Apatinib inhibited lung CSC -like properties via β-catenin. **a** Schematic diagram of lung tumor spheres culture after apatinib treatment with or without transfecion. A549 and H1299 cells were transfected with con siRNA, β-catenin siRNA, control vector, and pcDNA-β-catenin. After transfection, cells were treated with 5 μM apatinib for 3 days in SFM. **b**–**c** Protein level of β-catenin in β-catenin siRNA or plasmid transfected spheroids after apatinib treatment. One-way ANOVA (Bonferroni’s multiple-comparison test) was used. Data are presented as mean ± SD (*n* = 3). **p < 0.01 compared to the con siRNA or vector group, ^#^*p* < 0.05, ^##^*p* < 0.01 compared to the apatinib (5 μM) group. **d** Sphere-forming capacities of cells transfected with con siRNA or β-catenin siRNA after apatinib treatment. Scale bar = 200 μm. One-way ANOVA (Bonferroni’s multiple-comparison test) was used. Data are presented as mean ± SD (*n* = 3). *p < 0.05, **p < 0.01 compared to the con siRNA group. ^##^*p* < 0.01 compared to the apatinib (5 μM) group. **e** Protein levels of lung CSC markers in β-catenin-suppressed spheroids after apatinib treatment. One-way ANOVA (Bonferroni’s multiple-comparison test) was used. Data are presented as mean ± SD (*n* = 3). **p < 0.01 compared to the con siRNA group, ^#^ *p* < 0.05 compared with the apatinib (5 μM) group. **f** Sphere-forming capacities of cells transfected with control vector or pcDNA-β-catenin after apatinib treatment. Scale bar = 200 μm. One-way ANOVA (Bonferroni’s multiple-comparison test) was used. Data are presented as mean ± SD (*n* = 3). *p < 0.05, **p < 0.01 compared to the vector group, ^##^*p* < 0.01 compared to the apatinib (5 μM) group. **g** Protein levels of lung CSC markers in β-catenin-overexpressed spheroids after apatinib treatment. One-way ANOVA (Bonferroni’s multiple-comparison test) was used. Data are presented as mean ± SD (*n* = 3). *p < 0.05, **p < 0.01 compared with the vector group, ^#^*p* < 0.05, ^##^*p* < 0.01 compared with the apatinib (5 μM) group. **h**–**i** Protein levels of drug resistance-related genes in β-catenin- overexpressed or suppressed lung cancer spheroids after apatinib treatment. One-way ANOVA (Bonferroni’s multiple-comparison test) was used. Data are presented as mean ± SD (*n* = 3). *p < 0.05, **p < 0.01 compared to the con siRNA or vector group, ^#^*p* < 0.05, ^##^*p* < 0.01 compared to the apatinib (5 μM) group.

Based on the important role of β-catenin in the maintenance of lung CSC-like properties and drug resistance, we postulated that β-catenin could mediate the inhibitory effects of apatinib. To test this hypothesis, we first examined whether knockdown or overexpression of β-catenin would affect the inhibitory effect of apatinib on lung CSCs (Fig. 5a). Figures 5b and 5c showed that 5 μM apatinib plus β-catenin knockdown led to a more significant decrease in β-catenin expression relative to apatinib alone, while β-catenin overexpression partially reversed the effect of apatinib. Moreover, compared to apatinib alone, apatinib plus β-catenin siRNA also resulted in a more significant decrease in sphere formation and the expression of lung CSC-specific markers and drug resistance-genes (Figs. 5d, e, and h), while these effects were partially reversed by β-catenin overexpression (Figs. 5g, f, and i). Together, these data suggested that the inhibitory role of apatinib on lung CSC-like properties was, at least in part, mediated by β-catenin suppression.

### The inhibitory effect of apatinib on β-catenin expression was associated with ROS production

**Fig. 6:**
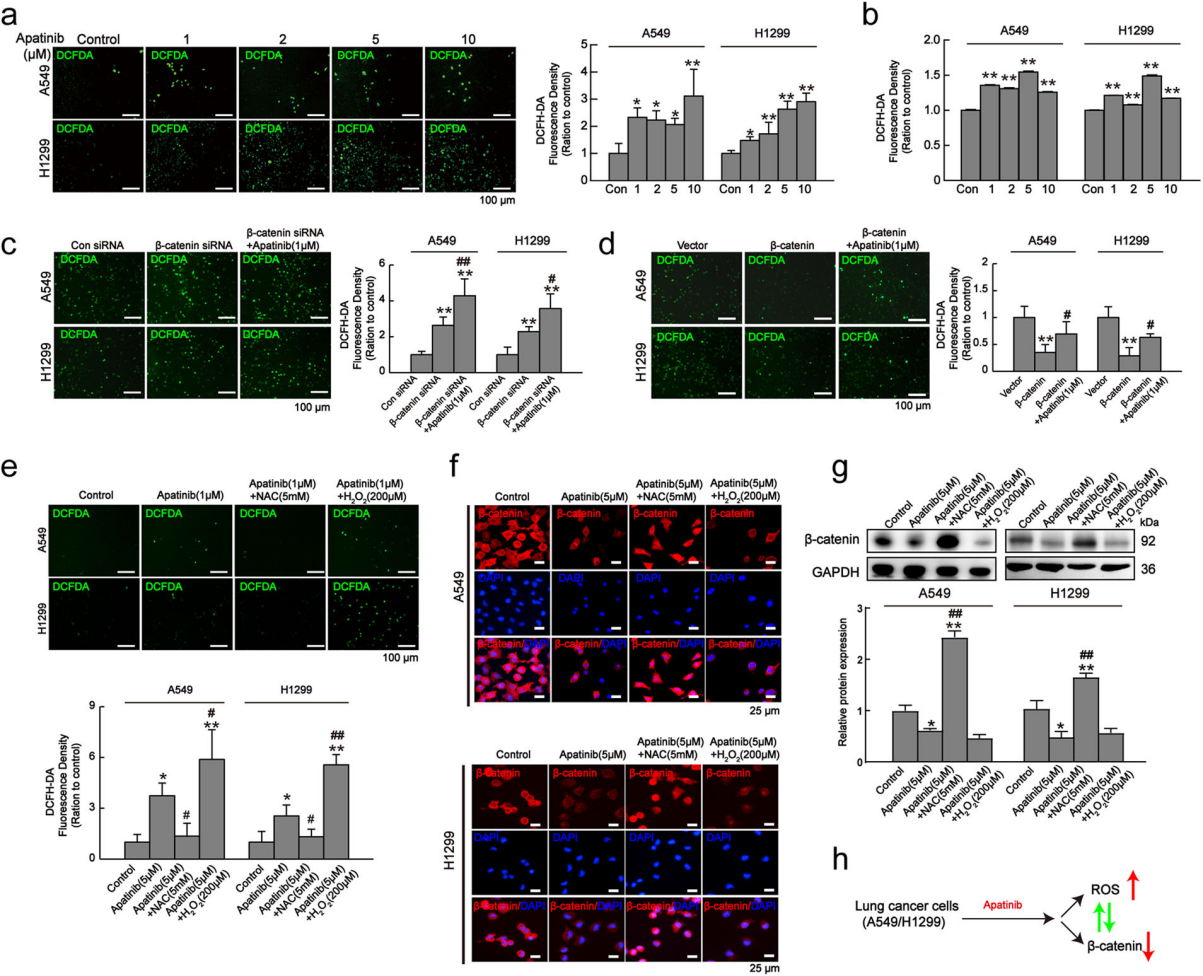
Apatinib induced ROS and downregulated β-catenin, leading to the suppression of lung CSC-like properties. **a** Immunofluorescence analysis of ROS in A549 and H1299 cells after various concentrations of apatinib treatment. Scale bar = 100 μm. One-way ANOVA (Bonferroni’s multiple-comparison test) was used. Data are presented as mean ± SD (*n* = 3). *p < 0.05, **p < 0.01 compared to the control group. **b** Flow cytometry analysis of ROS. One-way ANOVA (Bonferroni’s multiple-comparison test) was used. Data are presented as mean ± SD (*n* = 3). *p < 0.05, **p < 0.01 compared with the control group. **c** Immunofluorescence analysis of ROS in β-catenin siRNA-transfected A549 and H1299 cells treated with or without apatinib (1 μM) for 24 h. Scale bar = 100 μm. One-way ANOVA (Bonferroni’s multiple-comparison test) was used. Data are presented as mean ± SD (*n* = 3). **p < 0.01 compared to the control group; ^#^*p* < 0.05, ^##^*p* < 0.01 compared to the β-catenin siRNA group. **d** Immunofluorescence analysis of ROS in β-catenin-transfected cells treated with or without apatinib (1 μM) for 24 h. Scale bar = 100 μm. One-way ANOVA (Bonferroni’s multiple-comparison test) was used. Data are presented as mean ± SD (*n* = 3). **p < 0.01 compared to the control group; ^#^*p* < 0.05 compared with the β-catenin group. **e** Immunofluorescence analysis of ROS in apatinib-treated cells treated with 5 mM NAC or 200 μM H_2_O_2_ for 24 h. Scale bar = 100 μm. One-way ANOVA (Bonferroni’s multiple-comparison test) was used. Data are presented as mean ± SD (*n* = 3). *p < 0.05, **p < 0.01 compared to the control group; ^#^*p* < 0.05, ^##^*p* < 0.01 compared to the apatinib (1 μM) group. **f** Immunofluorescence analysis of β-catenin (red) expression in apatinib-treated cells with/without 5 mM NAC or 200 μM H_2_O_2_ for 24 h. DAPI: blue. Scale bar = 25 μm. **g** Protein level of β-catenin in apatinib (5 μM)-treated cells with/without 5 mM NAC or 200 μM H_2_O_2_. One-way ANOVA (Bonferroni’s multiple-comparison test) was used. Data are presented as mean ± SD (*n* = 3). *p < 0.05, **p < 0.01 compared to the control group; ^##^*p* < 0.01 compared to the apatinib (5 μM) group. **h** Schematic representation of the effect of apatinib on β-catenin and ROS in lung cancer cells.

Lower levels of ROS were found in CSCs compared to those in non-CSCs that preserve stemness^31^, which makes the regulation of ROS in the tumor microenvironment of CSCs a valid cancer treatment method. Previous studies have reported that apatinib induced ROS in pancreatic cancer^32^, which was also confirmed in the present study. Figure 6a showed that ROS production significantly increased after apatinib treatment for 24 h. Given that the maximum plasma concentration (C_max_) of apatinib after oral administration was achieved in 3 or 4 h^33^, Flow cytometry analysis further showed that 3 h of apatinib treatment already promoted ROS production. Notably, a significant increase in ROS production with 1 μM apatinib was also observed (Fig. 6b).

Previous studies found that ROS suppressed the activation of β-catenin pathway^34^, and we investigated whether apatinib could exert its effect on lung CSCs by promoting the ROS-mediated β-catenin downregulation. We observed that ROS production was significantly increased in β-catenin siRNA-transfected A549 and H1299 cells, whereas the opposite effects were observed in pcDNA-β-catenin-transfected cells. Moreover, a more prominent increase or decrease in ROS production with β-catenin siRNA or pcDNA-β-catenin plus 1 μM apatinib was also observed (Figs. 6c and d). These results suggested that apatinib not only directly promoted ROS production, but also suppressed β-catenin to produce ROS.

To further illustrate whether apatinib-increased ROS also negatively regulated β-catenin expression, we incubated lung cancer cells with apatinib plus N-acetyl-L-cysteine (NAC) or H_2_O_2_. As shown in Fig. 6e, apatinib (1 μM) caused a significant increase in ROS production, which could be reversed by NAC but enhanced by H_2_O_2_. Since, our above results showed that 5 μM apatinib caused a significant decrease in β-catenin (Fig. 3e), therefore, we focused our analysis on 5 μM apatinib plus NAC or H_2_O_2_ to assess whether ROS scavenge or overproduce could regulate β-catenin expression. As expected, our results showed that NAC reversed the decrease in β-catenin by apatinib, while H_2_O_2_ further enhanced the effects of apatinib (Figs. 6f and g), suggesting that the increase in cellular ROS caused by apatinib treatment also inhibited β-catenin.

Taken together, these results suggested that apatinib exerted an inhibitory role in lung CSCs by regulating ROS generation and β-catenin, and the interaction between ROS and β-catenin could further enhance the suppressive effects of apatinib (Fig. 6h).

### Apatinib disrupted redox balance and mitochondrial function, leading to the suppression of lung CSC-like properties

**Fig. 7:**
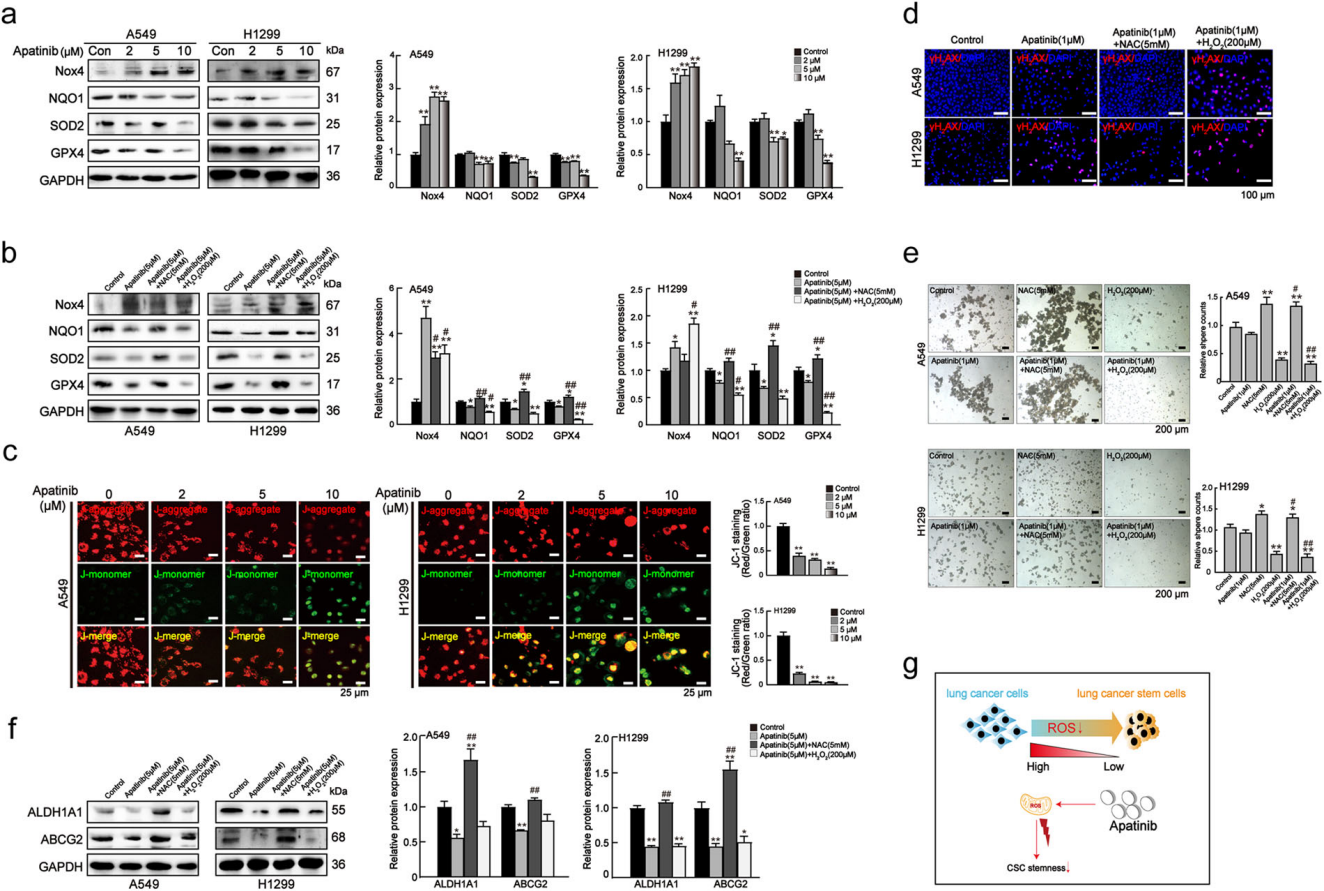
Apatinib disrupted redox balance and mitochondrial function to suppress lung CSC-like properties. **a** Protein levels of NOX4, NQO1, SOD2, and GPX4 in A549 and H1299 cells after various concentrations of apatinib treatment. One-way ANOVA (Bonferroni’s multiple-comparison test) was used. Data are presented as mean ± SD (*n* = 3). *p < 0.05, **p < 0.01 compared to the control group. **b** Protein levels of NOX4, NQO1, SOD2, and GPX4 in apatinib (5 μM)-treated cells treated with/without 5 mM NAC or 200 μM H_2_O_2_ for 48 h. One-way ANOVA (Bonferroni’s multiple-comparison test) was used. Data are presented as mean ± SD (*n* = 3). *p < 0.05, **p < 0.01 compared to the control group; ^#^*p* < 0.05, ^##^*p* < 0.01 compared to the apatinib (5 μM) group. **c** Immunofluorescence analysis of JC-1 in cells after various concentrations of apatinib treatment for 24 h. Scale bar = 25 μm. One-way ANOVA (Bonferroni’s multiple-comparison test) was used. Data are presented as mean ± SD (*n* = 3). **p < 0.01 compared to the control group. **d** Immunofluorescence analysis of γH_2_AX (red) in apatinib (1 μM) -treated cells with/without 5 mM NAC or 200 μM H_2_O_2_. DAPI: blue. Scale bar = 100 μm. **e** Sphere formation assays of apatinib (1 μM)-treated with/without 5 mM NAC or 200 μM H_2_O_2_. Scale bar = 100 μm. One-way ANOVA (Bonferroni’s multiple-comparison test) was used. Data are presented as mean ± SD (*n* = 3). *p < 0.05, **p < 0.01 compared to the control group; ^#^*p* < 0.05, ^##^*p* < 0.01 compared to the apatinib (1 μM) group. **f** Protein levels of ALDH1A1 and ABCG2 in apatinib (5 μM) treated with/without 5 mM NAC or 200 μM H_2_O_2_ 48 h. One-way ANOVA (Bonferroni’s multiple-comparison test) was used. Data are presented as mean ± SD (*n* = 3). *p < 0.05, **p < 0.01 compared to the control group; ^##^*p* < 0.01 compared with the apatinib (5 μM) group. **g** Schematic representation of the effects of apatinib on lung CSC-like properties by inducing redox imbalance.

Next, we analyzed the mechanisms underlying the ROS increase in apatinib-treated lung cancer cells. First, we focused on NADPH oxidase 4 (NOX4), a major enzyme contributing to ROS production in NSCLC^35^. Western blot analysis revealed that apatinib significantly increased NOX4 expression (Fig. 7a). Next, we verified whether the increased ROS caused by apatinib was associated with decreased antioxidant enzymes. Among these enzymes, NAD(P)H: quinone oxidoreductase 1 (NQO1), superoxide dismutase-2 (SOD2), and glutathione peroxidase 4 (GPX4) are highly expressed in lung cancer cells^36^ and are associated with drug resistance. As shown in Fig. 7a, apatinib significantly suppressed NQO1, SOD2, and GPX4 expression. Furthermore, NAC treatment in apatinib-treated cells substantially restored these enzymes expression, while H_2_O_2_ partially enhanced the effects of apatinib (Fig. 7b). These results suggested that apatinib disrupted the redox balance to elevate ROS in lung cancer.

Excessive ROS can directly disrupt the mitochondria by lowering the mitochondrial membrane and breaking the DNA helix. Previous studies have found that apatinib disrupts the mitochondrial transmembrane potential in leukemia cells^37^. To determine whether apatinib-increased ROS could directly influence mitochondrial function, we stained lung cancer cells with JC-1 to examine the mitochondrial membrane potential (MMP). With the increase in apatinib concentration, the green fluorescence level of the J-monomer was increased, while the red fluorescence level of the J-aggregates was decreased, which suggested that apatinib-triggered ROS induced MMP reduction (Fig. 7c). Subsequently, we investigated the influence of apatinib on DNA damage repair by assessing γH_2_AX (at ser^139^) induction, which is widely used as a marker of DNA damage and repair marker. Figure 7d showed that 1 μM apatinib increased the percentage of γH_2_AX positive cells. When incubated with NAC or H_2_O_2_, it was found that NAC treatment dramatically reversed the effects of apatinib, whereas H_2_O_2_ caused a significant increase in γH_2_AX positive cells.

The extent of DNA damage has an important influence on the fate of CSCs. We found that 1 μM apatinib reduced sphere formation, although the difference was not statistically significant (Fig. 7e), suggesting that the increase in cellular ROS caused by 1 μM apatinib did not reach the extent to which it could damage CSCs. After NAC treatment, the sphere formation ability was dramatically enhanced, while H_2_O_2_ treatment almost completely inhibited sphere formation. Simultaneously, Fig. 7f showed that the inhibitory effects of 5 μM apatinib on ALDH1A1 and ABCG2 were reversed by NAC, however, no change was observed after H_2_O_2_ treatment.

These results suggested that low concentration apatinib increased ROS production, disturbed redox balance and induced mitochondrial dysfunction. With the increase in apatinib concentration, the direct inhibitory effects of apatinib on lung CSCs enhanced the effect of ROS, leading to the suppression of lung CSCs traits (Fig. 7g).

### Effect of apatinib on in vivo tumor formation

**Fig. 8:**
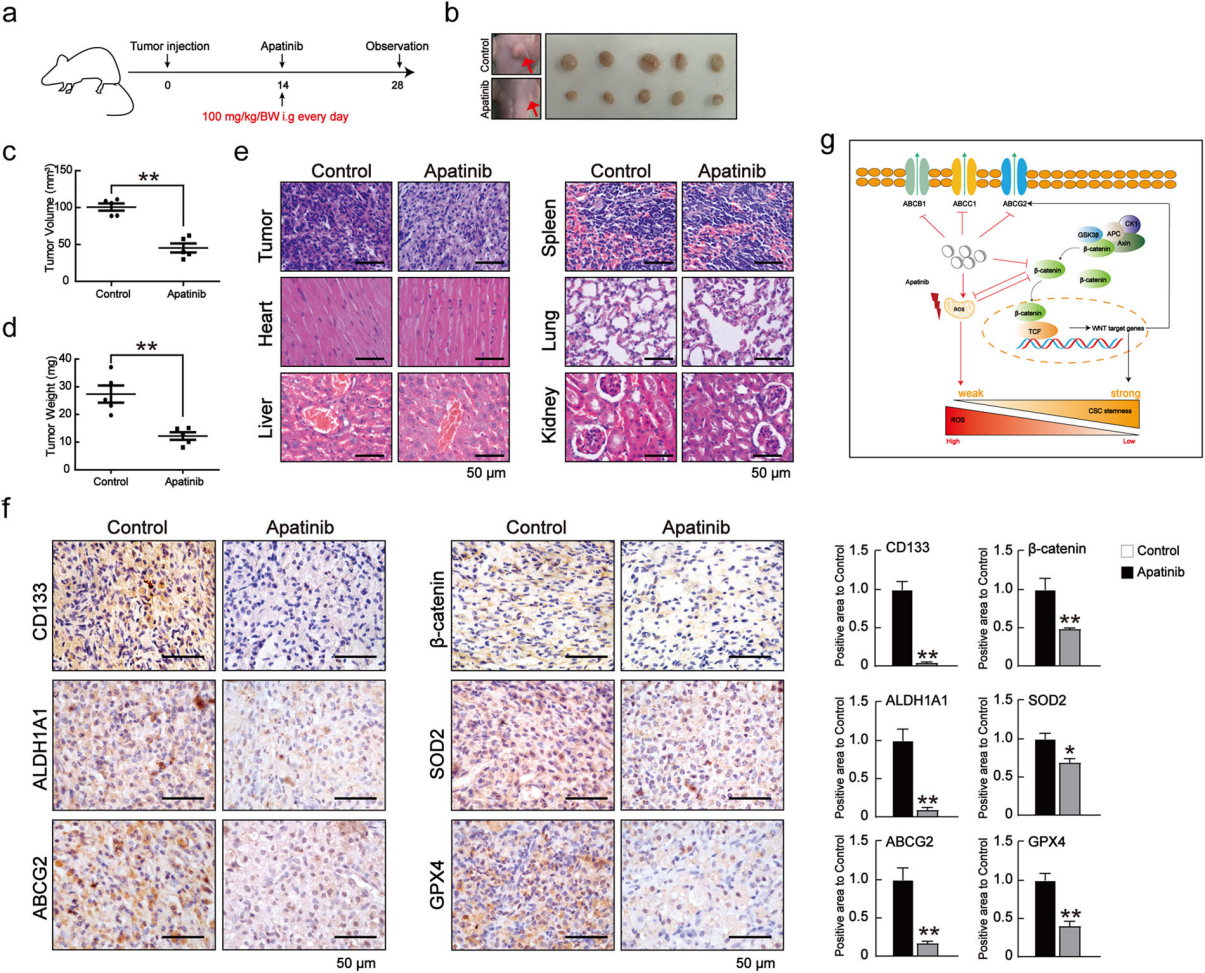
Apatinib inhibited tumor growth in vivo. **a** Male BALB/c nude mice (5 weeks of age) were injected with 5 × 10^6^ A549 cells. After 14 days, apatinib (100 mg/kg body weight) was orally administrated daily for another 14 days. **b** Subcutaneous tumors in mice on the 28th day after the injection of A549 cells. **c**–**d** Tumor volumes and masses were determined on the 28th day after injection. Unpaired *t*-test was used. Data are presented as mean ± SD (*n* = 5). **p < 0.01 compared to the control group. **e** H&E staining of tumor, heart, liver, spleen, lung and kidney. Scale bar = 50 μm. **f** Immunohistochemical staining of CD133, ALDH1A1, ABCG2, β-catenin, SOD2, and GPX4 expression in the xenograft tumors derived from the control group and the apatinib-treated group. Scale bar = 50 μm. Unpaired *t*-test was used. Data are presented as mean ± SD (*n* = 5). *p < 0.05, **p < 0.01 compared to the control group. **g** The representative working model of the study. Apatinib directly inhibited lung CSC-like properties by targeting Wnt/β-catenin signaling and decreasing lung CSC-specific markers; apatinib disturbed redox balance to induce ROS generation; ROS production and β-catenin suppression interacted with each other, leading to the inhibition of lung CSC stemness.

Based on the observation that apatinib potently suppressed lung CSC-like properties in vitro, and the fact that CSCs are a subpopulation of tumor cells with high tumorigenicity in vivo, we further examined the effect of apatinib on the tumor formation in vivo. A549 cells (5 × 10^6^) were inoculated into BALB/c nude mice by subcutaneous injection, and apatinib (100 mg/kg body weight) was orally administrated daily (Fig. 8a). After administration for 14 days, apatinib significantly inhibited tumor size and volume (Figs. 8b–d). Furthermore, the structures of the tumor, heart, liver, spleen, lung, and kidney were examined by haematoxylin and eosin (H&E) staining (Fig. 8e). The results showed that compared to the control group, apatinib-treated tumors had markedly fewer tumor cells, whereas the other tissues showed no significant change, suggesting that the dose of apatinib in the study did not exhibit obvious toxicity and appropriate for the treatment of lung cancer. Immunohistochemistry further confirmed that apatinib significantly decreased the expression levels of CD133, ALDH1A1, ABCG2, β-catenin, SOD2, and GPX4 (Fig. 8f). These data revealed that apatinib significantly inhibits the tumorigenicity of lung cancer cells in vivo, which was associated with the anti-lung CSC-like properties of apatinib (Fig. 8g).

## Discussion

Advanced and refractory/recurrent NSCLC is the leading cause of death in lung cancer patients, thus requiring the development of novel treatment strategies. Apatinib exhibits promising efficacy and manageable toxicity in patients with lung cancer, particularly in heavily treated, chemoresistant, or metastatic lung cancer patients^38,39^. Although numerous studies have highlighted the encouraging antitumor efficacy of apatinib against NSCLC, the underlying mechanisms remains poorly understood. In this study, we demonstrated that apatinib directly impeded β-catenin signaling and induced an increase in cellular ROS, led to the suppression of lung CSC phenotype.

The presence of lung CSCs is thought to be the root of tumor development, which are refractory to standard therapy and are closely related to metastasis, drug resistance, and tumor recurrence. By using a sphere formation system^40^, we showed that apatinib inhibited sphere formation and downregulated the expression of lung CSC-specific markers (CD133, CD44, ALDH1A1, Nanog, Oct4, and Sox2), suggesting the effective suppression of apatinib on lung CSCs. Meanwhile, we found a similar decrease of drug-resistant genes (ABCB1, ABCC1, and ABCG2) in lung cancer spheroids after apatinib treatment, which was further confirmed in CDDP-resistant A549 cells. Our data indicated that apatinib inhibited the acquisition of chemoresistance by decreasing cellular efflux. Therefore, apatinib affected lung CSCs by directly targeting lung CSC-specific markers.

The development and maintenance of CSCs stemness is closely associated with aberrant Wnt signaling, a critical signal activated in NSCLC^41^. β-catenin was also found significantly increased in CDDP-selected A549 cells^11^. Our results demonstrated that apatinib significantly downregulated the expressions of β-catenin, c-Myc, and cyclin D1 in lung CSCs. Hence, β-catenin suppression contributed to the inhibitory effects of apatinib on lung CSCs. However, β-catenin overexpression only partially reversed the inhibitory effects of apatinib on lung CSC markers and drug-resistance genes. Therefore, other mechanisms might be involved in the action of apatinib on lung CSCs and need to be explored.

Cancer cells exhibit high levels of ROS, which not only promote tumor development and progression, but also render them close to the death threshold of oxidative stress. Most chemotherapeutics induce ROS generation in cancer cells^42^. Therefore, we speculated that the inhibitory effects of apatinib on lung cancer are also related to ROS production. Numerous studies have demonstrated that apatinib alone or in combination with other chemotherapies has been shown as an effective and tolerable treatment option for a variety of cancers^43,44^. For example, Zhao et al. found that apatinib combined with PD-1/PD-L1 blockade therapy induced an enhanced therapeutic effect in lung cancer^18^. However, the precise underlying mechanism remains unclear. In this study, we revealed that 1 μM apatinib induced a significant increase in intracellular ROS in a short period of time. Currently, numerous clinical studies have demonstrated that apatinib combined with chemotherapy augment the sensitivity of advanced or refractory lung cancer, probably due to apatinib-induced ROS production that modulates the tumor microenvironment. However, the mechanism by which apatinib-induced ROS production has not yet been clarified.

Previous studies have demonstrated that ROS negatively regulates β-catenin expression through its degradation in lung cancer^45^. It is unclear whether apatinib-induced ROS production further decrease β-catenin expression. Our data demonstrated that apatinib increased ROS production, while reducing β-catenin expression. Meanwhile, β-catenin inhibition further induced ROS production, which consequently hindered β-catenin expression. Therefore, apatinib exhibited an anti-CSC action through crosstalk between ROS production and β-catenin repression. NOXs have been shown to be the key sources of ROS in mammalian cells. Among them, NOX4 is the most frequently overexpressed isoform in cancer cells and has been shown to promote NSCLC cell proliferation and metastasis^35^. We found that apatinib significantly increased NOX4 expression, which suggested that NOX4 participated in apatinib-induced ROS production in lung cancer cells.

Compared to healthy cells, cancer cells and CSCs often display enhanced antioxidant systems, which protect cancer cells against oxidative stress and provide CSCs or therapy-resistant cells with improved tolerance to drug stress. For example, advanced and treatment-resistant NSCLC cases exhibit NQO1 overexpression^46^. Elevated level of SOD2, the primary mitochondrial oxidative scavenger, was found in several cancer cells. It is known that upregulated SOD2 promotes cancer stemness and reduces apoptosis by scavenging ROS, thus mediating resistance to chemotherapy; in contrast, SOD2 inhibition impedes cancer progression^47^. Elevated GPX4 expression has also been found in radioresistant cells^48^. GPX4 is capable of selectively reducing lipid hydroperoxidase and decreasing subsequent reactive carbonyl species (RCS) accumulation. RCS can be initiated by and reciprocally amplify ROS, both of which inhibit or kill cancer cells. Interestingly, ALDH can also counter RCS/ROS by upregulating the anti-RCS/ROS system, thus mediating the maintenance of lower levels of RCS/ROS in cancer cells. In the present study, we observed that apatinib reduced the expression levels of NQO1, SOD2, and GPX4, while NAC treatment reversed these effects. Moreover, we showed that NAC treatment also restored the expression of ALDH1A1 and ABCG2 in apatinib-treated lung cancer cells. Lei et al found that ALDH1A1 upregulated SOD2, GPX4, and itself to mitigate erlotinib-induced ROS/RCS and confer drug resistance in lung adenocarcinomas^49^. Therefore, these results suggested that ROS production in apatinib-treated lung cancer cells was related to the inactivation of these antioxidant enzymes, which further inhibited lung CSCs.

Chemotherapeutic drugs generate multiple types of ROS that induce severe DNA damage in cancer cells. For example, CDDP induces a mitochondrial-ROS response that contributes to cytotoxicity^50,51^. Our resultes showed that apatinib caused mitochondrial dysfunction and increased the level of the DNA damage marker γH_2_AX. Apatinib induced-γH_2_AX was reversed by NAC treatment, while enhanced by H_2_O_2_ treatment. Previous studies have demonstrated that apatinib alone had no impact on apoptosis and cell cycle distribution of A549/PTX (paclitaxel) cells, while apatinib in combination with PTX dramatically increased ROS levels in the cells^52^. Based on previous studies and our results, low dose apatinib augmented the sensitivity of drug resistant cells. Moreover, our results showed that apatinib (1 μM) alone had no impact on sphere formation, and the number of spheres was even increased after NAC treatment, whereas H_2_O_2_ almost completely hindered sphere formation. Taken together, these results suggested that apatinib exerted cooperative effects with chemotherapeutics, and augmented the sensitivity of drug-resistant cells.

In conclusion, our study demonstrated that apatinib directly inhibited lung CSC-like properties by targeting Wnt/β-catenin signaling and decreasing lung CSC-specific markers; apatinib disturbed redox balance to induce ROS generation; ROS production and β-catenin suppression interacted with each other, leading to the inhibition of lung CSC stemness. Our findings could provide better understanding of the anti-cancer mechanisms of apatinib in application in combating advanced and refractory/recurrent lung cancer.

## Materials and methods

### Cell culture and sphere formation assay

Human NSCLC cell lines A549 and H1299 were obtained from the Chinese Academy of Typical Culture Collection Cell Bank (Shanghai, China) and routinely maintained in RPMI 1640 (Gibco, Carlsbad, CA, USA), supplemented with 10% fetal bovine serum (Gibco, Carlsbad, CA, USA). Spheroids were cultured as previously described^53^. Cells were seeded in a 12-well ultralow plate at a density of 50,000 cells and cultured in a serum-free medium (SFM) [DMEM/F12 (Gibco, Carlsbad, CA, USA) containing 20 ng/mL EGF (PeproTech, Rocky Hill, NJ, USA), 20 ng/mL bFGF (PeproTech, Rocky Hill, NJ, USA) and 2% B27 (Gibco, Carlsbad, CA, USA)]. To analyze the effects of apatinb on the spheres formation, A549 and H1299 cells were pre-treatment with or without N-acetyl-L-cysteine (NAC)/H_2_O_2_ for 30 min, and then were exposed to apatinib at various doses (0, 1, 2, 5, 10, and 20 μM) for 7 days in SFM. 0.1% DMSO was used as the vehicle control. Apatinib was obtained from Hengrui Medicine Co. Ltd. (Jiangsu, China) and dissolved in dimethyl sulfoxide (DMSO, Sigma-Aldrich, St. Louis, USA). Images of representative fields were acquired, and the number of the spheroids was counted under a microscope (sphere diameters >50 μm were counted). At least three fields were counted, and the data were expressed as the relative sphere formation.

### Generation of cisplatin-resistant A549 cells (A549-DR)

To establish A549-DR, A549 cells were continuously exposed to escalating doses of cisplatin (CDDP; Sigma) over a period of 6 months according to the established protocol^29^. The established A549-DR cells were maintained in RPMI 1640 containing 2 μg/mL CDDP.

### Quantitative reverse transcription-polymerase chain reaction (qRT-PCR)

Total RNA was extracted from cells using TRIzol reagent (Invitrogen, Carlsbad, CA, USA), and cDNA was synthesized from 1 μg of the total RNA from each sample according to the manufacturer’s instructions (Applied Biological Materials, Canada). qRT-PCR assays were performed using the EvaGreen 2×qPCR MasterMix (Applied Biological Materials) using a LightCycler96 real-time PCR detection system (Roche, Basel, Switzerland). The PCR primers for CD133, CD44, ALDH1A1, Nanog, Oct4, Sox2, ABCB1, ABCC1, ABCG2, β-catenin, c-Myc, and cylcin D1 were synthesized by Tsingke Biological Technology (Beijing, China) and the sequences were listed in Supplementary Table1. Data were normalized to GAPDH as an internal control and the fold changes in gene expression were derived using the 2^−ΔΔCt^ method. Each sample was analyzed at least thrice.

### Western blot analysis

Cells and spheroids were collected, lysed, and the protein levels were determined. Equal amounts of protein were separated by sodium dodecyl sulphate (SDS)-polyacrylamide gel electrophoresis and transferred to nitrocellulose membranes. The membranes were incubated with the appropriate primary antibodies overnight at 4 °C and then incubated with horseradish peroxidase-conjugated (HRP)-conjugated secondary antibodies for 1 h. Primary antibodies against CD133 (cat. no. 18470-1-AP, Proteintech, Rosemont, IL, USA), CD44 (cat. no. 15675-1-AP, Proteintech), ALDH1A1 (cat. no. 15910-1-AP, Proteintech), Nanog (cat. no. 14295-1-AP, Proteintech), Oct4 (cat. no. 60242-1-AP, Proteintech), Sox2 (cat. no. 11064-1-AP, Proteintech), β-catenin (cat. no. 51067-2-AP, Proteintech), c-Myc (cat. no. 10828-1-AP, Proteintech), cyclin D1 (cat. no. 26939-1-AP, Proteintech), ABCB1 (cat. no. 13342, Cell Signaling Technology, Boston, MA, USA), ABCC1 (cat. no. 14685, Cell Signaling Technology), ABCG2 (cat. no. 4477, Cell Signaling Technology), NOX4 (cat. no. bs-4730R, Bioss, Beijing. China), NQO1 (cat. no. 11451-1-AP, Proteintech), GPX4 (cat. no. ab125066, Abcam, Cambridge, MA, USA), SOD2 (cat. no. 13141, Cell Signaling Technology, Danvers, MA, USA), and GAPDH (cat. no. AP0063, Bioworld, Beijing, China) were used. The densitometric quantification of the protein bands were quantified using ImageJ software. GADPH as an internal loading control was used for normalization.

### Immunofluorescence staining assay

The cells or spheroids were fixed, permeabilized, incubated with 5% bovine serum albumin for 1 h, and then incubated with primary antibodies against CD133 (1:100 dilution, Proteintech), CD44 (1:200 dilution, Proteintech), β-catenin (1:200 dilution, Proteintech), ABCG2 (1:100 dilution, Cell Signaling Technology) and γH_2_AX (1:200 dilution, Cell Signaling Technology) overnight at 4 °C. After washing with PBS, cells or spheroids were incubated with FITC or Cy3-conjugated secondary antibody (Beyotime, China) for 1 h at 37 °C. Finally, the nuclei were stained with DAPI (Beyotime, China) for 5 min and images were acquired using confocal microscopy.

### Measurement of ROS

Intracellular ROS generation was assessed using 2′,7′-dichlorodihydrofluorescein diacetate (DCFDA; Sigma) according to the manufacturer’s instructions. Briefly, A549 and H1299 cells were washed twice with PBS and then incubated with fresh medium supplemented with 5 mM DCFDA at 37 °C for 20 min in the dark. DCF fluorescence intensity was detected using flow cytometry (Becton Dickinson, USA) and fluorescence microscopy (Nikon, Japan). The average fluorescence intensity was assessed with the ImageJ software.

### Transient transfection

β-catenin siRNA and control siRNA were purchased from Santa Cruz Biotechnology (Santa Cruz, CA, USA). pcDNA-β-catenin and control vector were purchased from Addgene (Cambridge, MA, USA). A549 and H1299 cells were cultured in 6-well plates at a density of 10^5^ cells in PRMI1640 medium. After incubation for 12 h, the cells were transiently transfected with pcDNA-β-catenin (2 μg), control vector (2 μg), or β-catenin siRNA (50 nM), and control siRNA (50 nM), using lipofectamine 3000 reagent (Invitrogen, Carlsbad, CA, USA) according to the manufacturer’s instructions. After transfection for 4–6 h, cells were collected and plated in SFM for another 3 days with or without apatinib.

### Mitochondrial membrane potential assay

After treatment with different concentrations of apatinib (0, 2, 5, and 10 μM) for 24 h, A549 and H1299 cells were stained with JC-1 from a Mitochondria Staining Kit (Beyotime) according to the manufacturer’s protocol. The cells were then analyzed using a fluorescence microscope (Nikon, Japan). The average fluorescence intensity was assessed with the ImageJ software and the red/green fluorescence ratio was determined.

### Evaluation of in vivo tumorigenicity

To test the effect of apatinib on the tumorigenic capacity, A549 cells were injected subcutaneously into mice (5 × 10^6^ cells/100 μL per flank). Fourteen days after injection, the mice were randomly assigned to two groups (*n* = 5 per group). Apatinib (100 mg/kg body weight) was administered daily by oral gavage. A saline solution was used as a vehicle control. Bidimensional tumor measurements were recorded using a digital caliper. Fourteen days after apatinib treatment, the mice were euthanized and tumor, heart, liver, spleen, lung and kidney tissues were harvested for histochemistry or immunohistochemistry. The tumor volume was calculated using the following formula: [width^2^ × length]/2. BALB/c nude mice (4–5 weeks old, male) were obtained from the Animal Research Center of Nanjing Medical University. Animal care was conducted in accordance with institutional guidelines, and the mice studies were performed with the approval of the Animal Care and Welfare Committee of Nanjing Medical University (IACUC-1907001).

### Histochemical or immunohistochemical staining

For histochemistry or immunohistochemistry, the tissues were fixed in 4% paraformaldehyde (PFA), dehydrated in a series of graded ethanol solutions, embedded in paraffin, and cut into 5 μm sections using a rotary microtome (Leica, Germany). The sections were then used for subsequent H&E staining or immunofluorescence microscopy. The antibodies used for immunohistochemistry were rabbit anti-CD133 (1:100 dilution, Proteintech), rabbit anti-ALDH1A1(1:100 dilution, Proteintech), rabbit anti-ABCG2 (1:100 dilution, Cell Signaling Technology), rabbit anti-β-catenin (1:100 dilution, Proteintech), rabbit anti-SOD2 (1:100 dilution, Proteintech) and rabbit anti-GPX4 (1:100 dilution, Abcam). Images were analyzed using the ImageJ program.

### Statistical analysis

All statistical analyses were performed using the SPSS (version 16.0, SPSS, Inc., Chicago, IL, USA). All experiments were repeated at least three times and representative results were presented as mean ± SD. Unpaired two-tailed Student’s *t* tests were used to compare differences between two groups, while one-way analysis of variance (ANOVA) was used to compare differences among multiple groups. Statistical significance was set at *p* < 0.05.

## Supplementary information

Supplemental Table 1
