# Supplementary material for: Apatinib suppresses lung cancer stem-like cells by complex interplay between β-catenin signaling and mitochondrial ROS accumulation
Source: Cell Death Discov. 2021 May 12;7:102. doi: 10.1038/s41420-021-00480-6 (PMC8115647; doi:10.1038/s41420-021-00480-6)
Supplement: Supplementary file 1 — Supplemental Table 1 [file 41420_2021_480_MOESM1_ESM.docx]

**Supplemental Table 1: RT-PCR primer sequence**

| Gene | Forward primer (5’-3’) | Reverse primer (5’-3’) |
| --- | --- | --- |
| CD133 | TACAACGCCAAACCACGACTGT | TCTGAACCAATGGAATTCAAGACCCTTT |
| CD44 | GACACATATTGTTTCAATGCTTCAGC | GATGCCAAGATGATCAGCCATTCTGGAAT |
| ALDH1A1 | GCACGCCAGACTTACCTGTC | CCTCCTCAGTTGCAGGATTAAAG |
| Nanog | GTTTGTGGGCCTGAAGAAAACT | AGGGCTGTCCTGAATAAGCAG |
| Oct4 | AGGGCTGTCCTGAATAAGCAG | CTGAATACCTTCCCAAATAGAACCC |
| Sox2 | CGAGTGGAAACTTTTGTCGGA | TGTGCAGCGCTCGCAG |
| ABCB1 | AAGCCACGTCAGCTCTGGAT | CTGCATTCTGGATGGTGGAC |
| ABCC1 | CACGACGCCTTCATGTTCTC | GGCTGGACAGGAGGAACAAC |
| ABCG2 | AGCAGCAGGTCAGAGTGTGG | CTGAAGCCATGACAGCCAAG |
| β-catenin | AAGACATCACTGAGCCTGCCAT | CGATTTGCGGGACAAAGGGCAA |
| c-Myc | GTCAAGAGGCGAACACACAAC | TTGGACGGACAGGATGTATGC |
| cylcin D1 | CGTGGCCTCTAAGATGAAGG | TGCGGATGATCTGTTTGTTC |
| GAPDH | CAAGGTCACCATGACAACTTTG | GTCCACCACCCTGTTGCTGTAG |
